# Telomere Q-PNA-FISH - Reliable Results from Stochastic Signals

**DOI:** 10.1371/journal.pone.0092559

**Published:** 2014-03-18

**Authors:** Andrea Cukusic Kalajzic, Nikolina Skrobot Vidacek, Miljenko Huzak, Milena Ivankovic, Ivica Rubelj

**Affiliations:** 1 Department of Molecular Biology, Ruder Boskovic Institute, Zagreb, Croatia; 2 Department of Mathematics, University of Zagreb, Zagreb, Croatia; University of Newcastle, United Kingdom

## Abstract

Structural and functional analysis of telomeres is very important for understanding basic biological functions such as genome stability, cell growth control, senescence and aging. Recently, serious concerns have been raised regarding the reliability of current telomere measurement methods such as Southern blot and quantitative polymerase chain reaction. Since telomere length is associated with age related pathologies, including cardiovascular disease and cancer, both at the individual and population level, accurate interpretation of measured results is a necessity. The telomere Q-PNA-FISH technique has been widely used in these studies as well as in commercial analysis for the general population. A hallmark of telomere Q-PNA-FISH is the wide variation among telomere signals which has a major impact on obtained results. In the present study we introduce a specific mathematical and statistical analysis of sister telomere signals during cell culture senescence which enabled us to identify high regularity in their variations. This phenomenon explains the reproducibility of results observed in numerous telomere studies when the Q-PNA-FISH technique is used. In addition, we discuss the molecular mechanisms which probably underlie the observed telomere behavior.

## Introduction

Telomeres are specialized structures at the ends of linear chromosomes, composed of repetitive DNA and an associated protein complex called shelterin [1]. They are dynamic structures, continuously losing their repeats with increasing cell divisions [2]. In normal somatic cells critically short telomeres signal growth arrest [3], [4] which is considered to be the main mechanism of senescence and consequently the process of aging. Telomere length is widely used as a reliable biomarker for longevity and aging related diseases, both at the individual and population level [5]–[7]. Since many authors draw conclusions about biological and medical phenomena based on these results, their accurate interpretation is a necessity. However, recent reports question the reproducibility and precision of Southern blot and quantitative polymerase chain reaction (Q-PCR), the two most common techniques used to follow telomere dynamics in experimental and epidemiological studies [8], [9]. While telomere length followed through Southern blot and Q-PCR analysis gives us plenty of information about their gross dynamics [10], it is very important to monitor the behavior of individual telomeres as well, especially when considering medical predictions or pharmaceutical effects [11], [12]. For these considerations, Q-PNA-FISH has become the method of choice.

**Figure 1:**
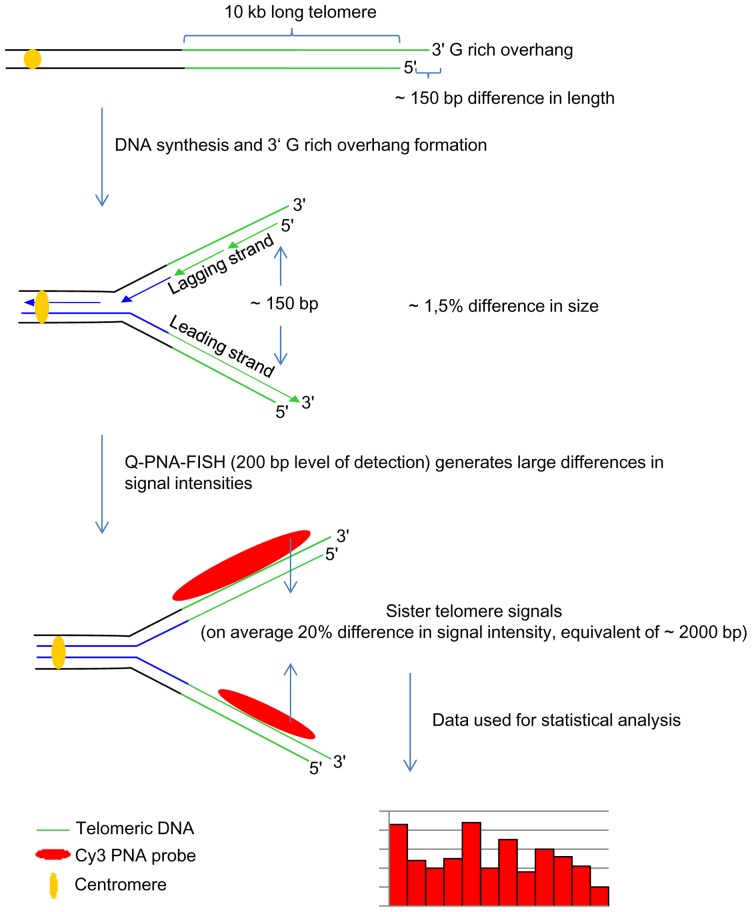
Schematic representation of telomere replication and subsequent Q-PNA-FISH labeling and analysis. For a 10∼1,5% of the longer sister length. Q-PNA-FISH labeling generates much larger differences among signal intensities. On average these differences are 20% which corresponds to about 2000 base pairs. We used measured signals for further mathematical and statistical modeling.

It has been established that the sensitivity level of Q-PNA-FISH is ∼200 bp [13]. Various techniques demonstrated that telomeres lose only about 50–150 base pairs per cell division which is below the detection level of Q-PNA-FISH (Figure 1) [2], [14], [15]. Thus, when metaphase chromosomes are analyzed, a time when sister telomeres are still together following replication, one could expect that their Q-PNA-FISH signal intensities will be about the same. However previously, we and others described great differences in Q-PNA-FISH signal intensities between sister telomere pairs in normal cells (Figure 1) [16]–[19]. Obviously this discrepancy is not a real biological phenomenon but the result of inefficient labeling of telomere repeat sequences by the PNA probe. This inefficiency in labeling results in, to some extent, random distribution of analyzed telomere Q-PNA-FISH signals. This is an important factor that one should keep in mind when interpreting the data in various studies. On the other hand numerous studies published to date showed consistent reproducibility in gross quantitative telomere Q-PNA-FISH results in various cell lines, chromosomes or different individuals [20]–[24]. Although this contradiction is of great importance for telomere research, no substantial effort has been made to provide a plausible explanation for it [17], [18]. Since telomere Q-PNA-FISH is still widely used in research studies and lately also in commercial analysis for the general population (lifelength.com, repeatdiagnostics.com), we decided to thoroughly address this inconsistency and performed extensive statistical analysis of sister telomere signal variations (Figure 1). We analyzed the relationship between Q-PNA-FISH signal intensities among sister telomeres and discovered a high correlation between the stronger telomere signal of the pair and difference variation of the corresponding sister telomere value. Our results points to the conclusion that there is a strong regularity in telomere signal variations obtained by Q-PNA-FISH and our statistical model is based on this finding. Also, this finding explains the reproducibility of results in numerous studies published to date which use Q-PNA-FISH for quantitative analysis of telomeres. In addition, we provide a model(s) for the obtained quantitative data, discuss technique reliability and point to probable molecular mechanisms that underlie the quantitative readings.

## Results

### Correlations between sister telomere signals show high regularity

**Figure 2:**
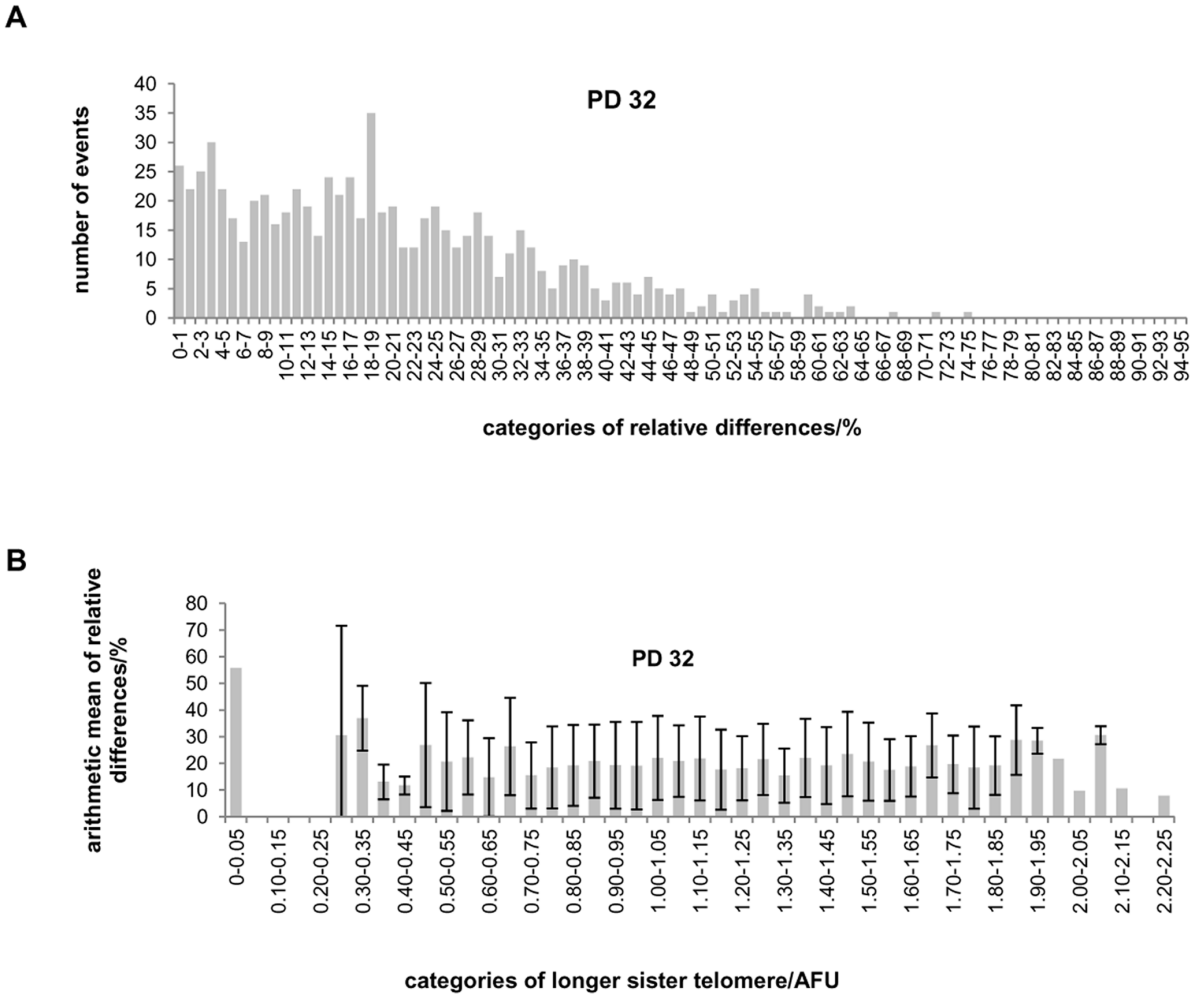
Telomeres of MJ90 cells. A) Distribution of telomere fluorescence signal relative differences between sister telomeres in MJ90 cells at PD 32. B) Arithmetic mean of relative differences between sister telomere signals in percentages in relation to longer telomere signals in MJ90 cells at PD32.

Q-PNA-FISH using a C rich probe for labeling of the G rich telomere strand is the most common technique used to follow individual telomere dynamics in numerous publications. We employed this method to analyze sister telomere pairs on metaphase chromosomes of normal and hTERT MJ90 human fibroblasts with increasing population doublings (PDs). Normal human fibroblasts have limited PDs at the end of which they enter senescence. With increasing PDs their telomeres continuously shorten, which may influence their conformation and consequently PNA probe hybridization dynamics. On the other hand, hTERT human fibroblasts MJ90 have telomeres that are maintained stably at constant length and presumably have a constant conformation. This will enable us to distinguish if results are influenced by changes in telomere length during cell senescence. The observed sister telomere signal intensities from each chromosome end are compared against each other. Absolute as well as relative differences with respect to longer sister signal intensity from the pair, are calculated. These values are used for subsequent graphical distribution and statistics. The data showed that distribution of relative differences varied between >1% and ≤75% for PD32, >1% and ≤62% for PD42 and >1% and ≤95% for PD52 respectively (Figure 2A and Figures S1A and S1B). For all PDs 70% of relative differences fall between ∼1 and 25%. Relative differences higher than 25% are rather rare and their frequencies decline even further with increasing PDs (Figure 2A and Figures S1A and S1B).

**Figure 3:**
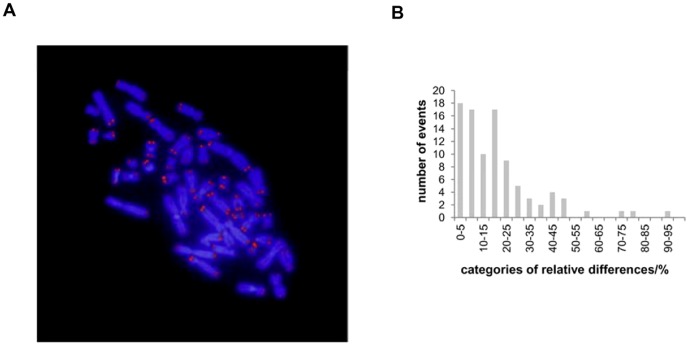
Example and analysis of one metaphase spread from MJ90 cells, PD 52. A) Merged image after *in situ* hybridization of telomere Cy3-PNA probe (red) on metaphase chromosomes stained with DAPI (blue). B) Distribution of relative differences between sister telomere signals in percentages for a given metaphase.

In addition to gross telomere statistics, this phenomenon is present at the individual cell level as well where relative differences follow the same distribution pattern with about the same mean value (Figures 3A and 3B). This means that the observed signal differences among sister telomeres obtained by Q-PNA-FISH are endogenous to all normal cells.

In order to reveal the relationship between relative difference and longer telomere sister signal intensity for each PD we portioned the data with respect to increasing categories of longer sister values. For each category the arithmetic mean and standard deviation of relative differences were calculated. We found that the obtained arithmetic means and standard deviations are about the same values regardless of categories for each PD (Figure 2B, Figures S2A and S2B). Arithmetic means group around 20.1% for PD 32, 19.7% for PD 42 and 18.1% for PD 52 respectively (Table 1) and standard deviations group around 0.14% for PD 32, 0.13% for PD 42 and 0.14% for PD 52 respectively (Table 2).

**Table 1:** Estimates of the parameter *α*.

MJ90	Sample size (*n*)	Mean rel. diff. ( 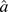 )	Std.dev.( 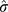 )	Std. error for *a* ( 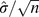 )	95% confidence interval for *a*
PD 32	742	0.201	0.145	0.005	[0.191, 0.211]
PD 42	379	0.197	0.133	0.007	[0.184, 0.211]
PD 52	423	0.181	0.144	0.007	[0.168, 0.195]

**Table 2:** Estimates of the parameter σ.

MJ90	Sample size (*n*)	Std. dev. ( 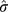 )	Kurtosis ( 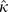 )	Std. error for logσ	95% confidence interval for σ
				( 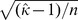 )	
PD 32	742	0.145	3.332	0.056	[0.137, 0.154]
PD 42	379	0.133	2.746	0.068	[0.124, 0.142]
PD 52	423	0.144	6.856	0.118	[0.129, 0.167]

**Figure 4:**
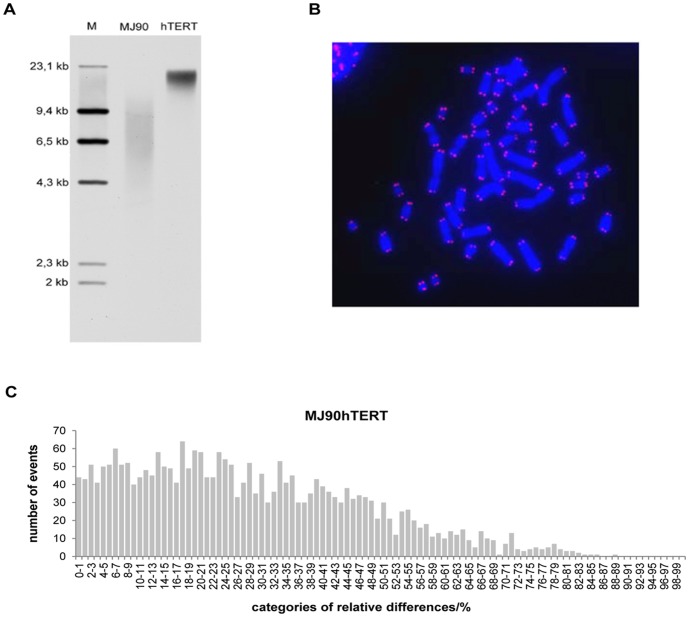
Telomeres of hTERTMJ90 cells, PD 127. A) Southern blot analysis of MJ90hTERT and MJ90 cells. *M* =  molecular weight marker. B) Q-PNA-FISH of MJ90hTERT cells. Merged image after *in situ* hybridization of telomere Cy3-PNA probe (red) on metaphase chromosomes stained with DAPI (blue). C) Distribution of telomere fluorescence signal relative differences between sister telomeres in MJ90hTERT cells.

This phenomenon is not specific only to normal cells which continuously lose their telomere repeats with increasing PDs but also applies to telomerase expressing cells. MJ90hTERT cells have constitutive expression of telomerase and unlike normal MJ90 cells, their telomeres are maintained at the longest narrow length range so that 90% of them fall between 16 and 18,5 kb with an average length of 17,5 kb (Figure 4A). One would not expect significant differences between sister telomere length in these cells. However, differences are widely present on metaphase Q-PNA-FISH spreads showing a distribution of relative differences among sister telomere signal intensities similar to normal MJ90 cells; between >1% and ≤90% with an average of 28% (Figures 4B and 4C).

**Figure 5:**
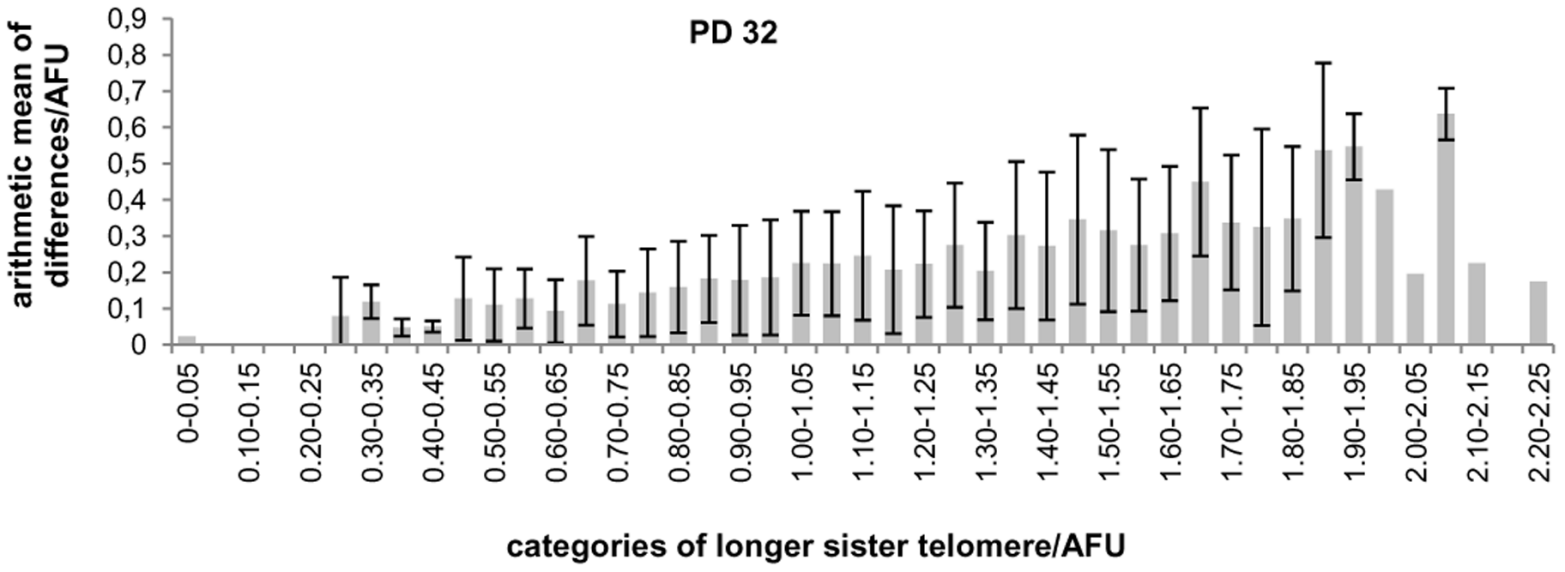
Arithmetic means of absolute differences with respect to longer telomere signals in MJ90 cells at PD32.

Our data on MJ90 fibroblasts show an obvious correlation between the arithmetic means of absolute differences among sister telomeres and the size of the longer telomere signal intensity from each pair such that the arithmetic means and standard deviations of absolute differences proportionally increase with the size of the longer telomere signal intensity (Figure 5 and Figures S3A and S3B). Therefore, the absolute differences are proportional to the signal intensities of the longer sister. This regularity is typical for all PDs. In the discussion we provide a possible explanation for telomere Q-PNA-FISH signal variations, but one can only speculate on the molecular mechanisms that ensure a proportional percentage of variations per telomere length. One possibility points to certain structural features of condensed telomeres. The reproducibility of data obtained by Q-PNA-FISH relies on this phenomenon.

### Statistical model and analysis

In order to perform an inferential analysis and comparison of sister telomeres belonging to populations with different PD's we propose a linear regression model of the form:
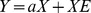
for the datum (*X*,*Y*) representing a sister telomere. Here *X* is the signal intensity of the sister with a longer telomere (actually, it is the maximum signal intensity between sister telomeres), and *Y* is the (absolute) difference between telomere signals of the sisters. In the model, *a* represents the mean relative difference between sister telomere signal intensities, the product *a·X* represents the expected difference between sister telomere signal intensities conditionally on a given telomere signal intensity *X* of the longer sister, and the error term *XE* is the random deviation between *Y* and *aX*. We assume that *XE* has conditional zero mean and standard deviation proportional to *X* for a given value of *X*, i.e. random variable *E* has zero mean and constant variance *σ* ^2^ conditionally with respect to the longer telomere signal intensity (see Section 1 in Data S1 for detailed description of the model). Hence the difference between sister telomere signals *Y* is a random variable with mean and standard deviation proportional to the signal *X* of the longer sister telomere conditionally on the longer sister telomere. The plausibility of the proposed model follows from the empirical regression functions of the absolute and relative differences between telomere sisters with respect to the signal intensity of the longer telomere sister based on the data obtained at PDs 32, 42, and 52 (Figures 2B and 5 and Figures S2A, S2B, S3A and S3B). Post analysis of the estimated models with standard verification methods shows that the proposed model provides a satisfactory description and prediction of the data in all cases (Section 3 in Data S1, Table S1, and standardized residuals at Figure S4).

Estimates of the model parameters *a* and *σ* obtained by the weighted least square method (expression (8) in Data S1) and their 95% confidence intervals (expressions (10) and (17) in Data S1) estimated by the bootstrap method (Data S1 Section 2) are presented in Tables 1 and 2. From the presented standard errors and sizes of 95% confidence intervals we can conclude that these parameters are estimated with satisfactory precision (relative standard errors for *a*: 2.5%–3.9%, and for *σ*: 6.2%–16.0%).

**Figure 6:**
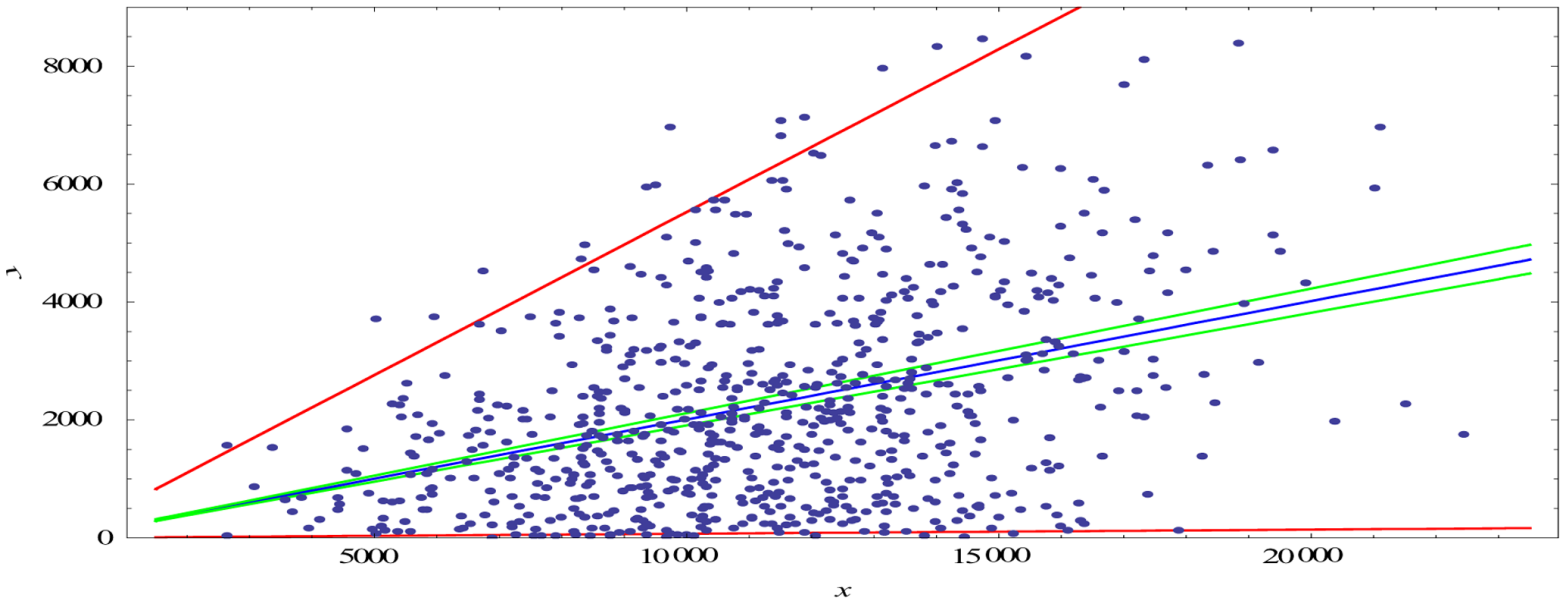
Estimated regression model for PD 32 sample: regression line (blue), 95% CIs of expected values of *Y* given *x* (ordinates of the green lines), and 95% CIs of the response values of *Y* given *x* (ordinates of the red lines).

Graphical comparison of the estimated models and 95% confidence intervals for predicting the conditional mean value of *Y* (expression (12) in Data S1) and new observation of *Y* (expression (15) in Data S1) with respect to a given value of *X*, with the data, are presented in Figure 6 and Figure S8. From these figures we can see that the model describes the data in all cases surprisingly well.

Although the sample sizes are fairly large we initially had to perform the bootstrap method for estimating the sample distributions for the appropriate pivot random quantities, and hence confidence intervals, because standardized residuals have a highly asymmetric distribution (see the right sides of Figure S6). It turned out that the bootstrap distributions of the pivot quantity for obtaining confidence intervals for parameter *a* (expression (9) in Data S1) in all considered populations do not significantly differ from the standard normal distribution (Figure S5) and hence the bootstrap approximation is not really needed in this case. The same quantity is a pivot for obtaining confidence intervals for predicting the mean value of *Y* given *X* (expression (11) in Data S1). Quite the opposite conclusion can be derived in the case of the pivot for obtaining confidence interval for predicting a new observation *Y* given *X* (expression (13) in Data S1): we can see from Figure S6 that the bootstrap distributions of the pivot look like a mirror image of the corresponding standardized residuals and hence cannot be approximated by the standard normal distribution in all cases. Such clear conclusions cannot be derived in the case of pivot (given by expression (16) in Data S1) for obtaining confidence intervals for error standard deviation *σ*. In the case of a population with PD 42 it seems that its bootstrap distribution does not significantly differ from standard normal but in other cases the distinction from normality is obvious (Figure S7).

For comparison of the corresponding model parameters between different populations (sister telomeres with different PDs) we performed the appropriate large sample z-tests (Data S1 Section 4, expressions (18) and (19)). The results are presented in Tables 3 and 4.

**Table 3:** Results of the one-sided z-tests for comparison *a*-parameters.

Hypothesis H_0_	Hypothesis H_1_	z-value	p-value
a_PD32_ = a_PD42_	a_PD32_ > a_PD42_	0.417	0.338
a_PD42_ = a_PD52_	a_PD42_ > a_PD52_	1.656	0.049
a_PD32_ = a_PD52_	a_PD32_ > a_PD52_	2.251	0.012

**Table 4:** Results of the two-sided z-tests for comparison *σ*-parameters.

Hypothesis H_0_	Hypothesis H_1_	z-value	p-value	p*-value
σ_PD32_ = σ_PD42_	σ_PD32_ ≠ σ_PD42_	1.989	0.047	0.050
σ_PD42_ = σ_PD52_	σ_PD42_ ≠ σ_PD52_	-1.175	0.240	0.228
σ_PD32_ = σ_PD52_	σ_PD32_ ≠ σ_PD52_	0.012	0.906	0.944

The mean relative difference between sister telomeres with PD 32 (*a*_PD32_) is not significantly greater than the same parameter with PD 42 (*a*_PD42_), and the mean relative difference between sister telomeres with PD 42 is not significantly greater than the same parameter for sisters with PD 52 (*a*_PD52_) just at 5% level of significance, but at the same level, *a*_PD32_ is significantly greater than *a*_PD52_. Therefore the mean relative differences have values of ∼20% or less, and slightly decrease with increasing PDs.

The error standard deviations of each pair of groups with different PDs are not significantly different at 5% level of significance. The same conclusion can be drawn by using normal approximations as well as bootstrap approximations of the null-distributions of the test-statistics (expression (19) in Data S1, and the 4^th^ and 5^th^ lower columns of Table 4). In the border case of comparison of the error standard deviations between groups with PD 32 and PD 42 the bootstrap-based *p*-value was needed to confirm the conclusion. Therefore we do not have enough evidence to conclude that error standard deviation *σ* changes its value with increasing PDs.

## Discussion

With respect to a gross telomere repeat loss of ∼50–150 bp per replication and a detection level of ∼200 bp with the Q-PNA-FISH method [13], sister telomeres in metaphase should show nearly identical fluorescence intensity. This is evidenced in experiments which use electron microscopy for direct identification of individual G-rich 3' overhangs in various cell lines and demonstrates that processes that take place in the maturation of a newly replicated telomere, including the formation of G-rich overhang, (during which ∼150–350 nucleotides is lost depending of the cell line)[15], cannot explain the differences in Q-PNA-FISH signals among sister telomeres which is usually, on the order of 1.5–5 kilobases. For MJ90 fibroblasts our quantitative analysis showed that for all PDs average relative differences among sister telomeres are substantial, grouping around 18–20% (Figure 1). Within small differences (∼150–350 nucleotides), sister telomere lengths are similar, but their Q-PNA-FISH signals are evidently stochastic as described by the statistical model in the manuscript.

Importantly, the range of these differences are not entirely random so that they proportionally increase with increasing signal intensity of the larger sister of the pair, indicating possible structural features that underlie this phenomenon. According to the presented results and previously published studies [20], [25] it is obvious that normal cells would not be able to reach the demonstrated PDs of approximately 55–60 as in the case of normal human MJ90 fibroblasts. An average telomere repeat loss of ∼20% per cell division could allow for just a few PDs. We should emphasize that abrupt telomere shortening [19], [26] observed in normal human fibroblasts cannot significantly contribute to observed differences between sister telomere signals since it occurs at very low frequency, estimated at ∼0.05% of all telomeres per PD [27]; Vidacek NS et al., unpublished results). Thus, it is obvious that the observed Q-PNA-FISH signals are artifacts to a certain point but we demonstrate and mathematically/statistically prove that this stochasticity is reproducible.

**Figure 7:**
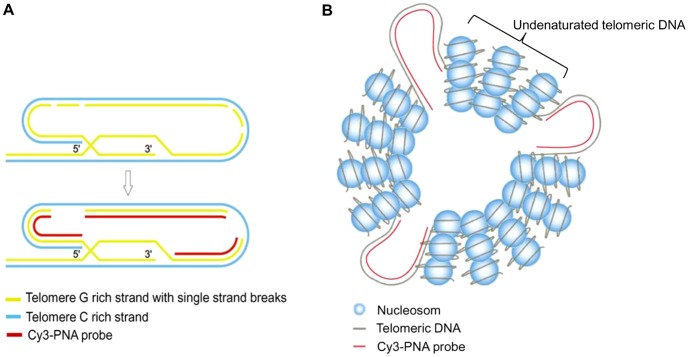
Models for telomere Q-PNA-FISH labeling. A) Single-stranded breaks along telomere G-rich strand and dissociation of stretches of the same strand could cause incomplete labeling of G-rich strand with PNA probe. B) Condensed telomere structures on metaphase chromosomes contribute to less effective labeling by Q-PNA-FISH.

Given the conclusion that the observed differences between sister telomeres are not real biological phenomenon but artifacts of the method we propose a couple of models to explain these findings and describe a combination of effects that may affect telomere Q-PNA-FISH hybridization based on known telomere structure and behavior. The first model is based on incomplete labeling of a G-rich strand with the PNA probe. It has been reported that telomere sequences are very susceptible to oxidative stress [28], [29] which induce single-stranded breaks in genomic DNA. The frequency of such breaks is significantly higher at telomere G-rich strands than elsewhere in the genome [29]. Such breaks are normally repaired within 24 hours but at telomeres they can stay unrepaired for up to twenty days [28]. One should keep in mind that during the preparation of metaphase chromosomes for Q-PNA-FISH analysis, aggressive chemicals, enzymes and conditions (HCl, formamide, pepsin, high temperature) are used. These can cause single-stranded breaks along telomere G-rich strands and dissociation of stretches of the same strand which could be washed off later in the procedure. Such telomeres would have a reduced hybridisation capacity and therefore reduced fluorescence intensity after hybridization with a Cy3-(C_3_TA_2_)_3_PNA probe (Figure 7A). Similarly, nicks and loss of stretches of the strand could happen on the C-rich strand as well but these would not influence Cy3-(C_3_TA_2_)_3_PNA probe labeling and quantitative results. Since all published data are obtained with Cy3-(C_3_TA_2_)_3_PNA probe on G-rich telomere strand we focused on effects related to this strand only.

Notably, following Q-PNA-FISH labeling, chromosomes do not disintegrate into relaxed DNA but maintain the integrity of their structure so that they can be positively identified in karyograms. Therefore, unlike PNA labeling of naked telomere sequences on plasmids, labeling of condensed telomere structures on metaphase chromosomes is much less effective relying on several important factors such as accessibility of proteinase to inner chromosome structures and efficiency of digestion, level of chromosome DNA denaturation which assume disassembly of nucleosomes and other higher order chromosomal structures and accessibility of PNA probes especially to inner parts of telomere (supra)structure (Figure 7B). Further, when relaxed, the telomere G-rich chain has a tendency to form a G-quadruplex [30] which has been positively identified on telomeres in mammalian cells [31]. Thus, PNA probes probably compete with endogenous G-quadruplex folding during renaturation which may also contribute to the observed unequal labeling among sister telomeres. We do not exclude that a combination of these (and perhaps some other) effects contribute to the observed Q-PNA-FISH labeling phenomenon. As far as we are aware, the proposed mechanism is the first attempt to provide a molecular explanation of the phenomenon and may serve as a concept for future experiments.

Our statistical analysis demonstrates that standard errors and 95% confidence intervals, for the mean loss per telomere signal intensity parameter throughout increasing PDs (Table 1 and Data S1) show high accuracy of the parameter estimations. From the data presented, we conclude that the mean difference per telomere signal intensity does not significantly change from PD 32 to PD 42 (*p*>.32), but the decrease in length of loss from PD 42 to PD 52 is significant (*p*<.05, see Table 3). Thus, when planning experiments with telomere Q-PNA-FISH we should keep in mind that younger normal cell cultures will demonstrate statistically more consistent results.

Although extensive statistical analysis demonstrated significant differences in the distribution of sister telomere signal intensities, we were able to prove a reproducible distribution of telomere sister ratios. High confidence in the observed variations among sister telomere Q-PNA-FISH signals ensures reproducibility of results in various studies employing this technique [5]–[7], [11], [12].

## Materials and Methods

### Cell Culture

Human diploid fibroblast strain MJ90 (HCA2) and MJ90hTERT (HCA2hTERT) was kindly provided by Dr Olivia M. Pereira-Smith (University of Texas, Health Science Center, San Antonio, TX, USA) [32], [33]. Cells were cultured in Dulbeco's modified Eagle's medium (DMEM; Sigma, St. Louis, MO, USA) supplemented with 10% fetal bovine serum (FBS; Gibco, Germany) at 37°C/5% CO_2_. The number of population doublings was determined at each subculture.

### Metaphase preparation and Q-PNA-FISH

Colcemid (0.1 mg/ml) was added to the cultures and cells were harvested 6 h later. After washing and hypotonic swelling, cells were fixed and stored in methanol/acetic acid fixative using standard procedures. Cells were fixed to slides by spinning small volumes (200 μl) of cells in 1 ml of methanol/acetic acid fixative. The slides were dried overnight in air and immersed in Phosphate Buffered Saline (PBS) for 5 min prior to fixation in 4% formaldehyde in PBS for 2 min, washes in PBS (3×5 min) and treatment with pepsin (P-7000, Sigma, St. Louis, MO, USA) at 1 mg/ml for 10 min at 37°C at pH 2.0. After a brief rinse in PBS, the formaldehyde fixation and washes were repeated and the slides were dehydrated with ethanol and air dried. Thirty microliters of hybridization mixture containing 70% formamide, 1.77 μM Cy3-(C_3_TA_2_)_3_ PNA probe (DACO, North America), 10% (W/V) blocking reagent (Roche, Indianapolis, IN, USA) 2 mM Tris-Cl pH 7.4, MgCl_2_ buffer (82 mM NaH_2_PO_4_/9 mM citric acid/20 mM MgCl_2,_ pH 7.4) in miliQ water was added to the slide, a coverslip (60×20 mm) was added and DNA was denatured by heat for 3 min at 80°C. After hybridization for 2 h at room temperature, the slides were washed at room temperature with 70% formamide/10 mM Tris-Cl pH 7.4/0.1% (W/V) BSA (2×15 min) and with 10 mM Tris-Cl pH 7.4/0.15 M NaCl pH 7.4 containing 0.1% Tween-20 (3×5 min). The slides were then dehydrated with ethanol, air dried chromosomes were counterstained with 0.1 mg/ml of 4,6-diamidino-2 phenylindoledehydrochloride (DAPI). The slides were then washed in dH_2_O, air dried and covered by a drop of antifade mounting media.

### Telomere length analysis

After PNA hybridization, fluorescence signals were visualized under a fluorescence microscope BX51 (Olympus, Japan) equipped with a filter wheel. After localization of metaphases, DAPI and Cy3 fluorescence signals were captured by a CCD camera (Olympus DP70, Japan), using FotoCanvas Lite v1.1 Software. Black and white images were used for quantitative analysis using Image-Master VSD software (Amersham Biosciences, UK). The mean pixel value of the background was subtracted from the pixel value of each telomere in the metaphase. Relative intensities of individual telomeres were obtained by dividing the mean pixel value of each telomere in the metaphase by the mean pixel value of all telomeres in the metaphase.

### Southern Blot Analysis

Genomic DNA was isolated with DNeasy Tissue Kit (Qiagen, Valencia, CA, USA) and digested with *Rsa* I/*Hin*f I (Roche, Indianapolis, IN, USA) restriction enzymes. Equal amounts (5 μg) of DNA were loaded on 0.8% agarose gel. Gel was depurinated, denatured, neutralized and DNA was transferred to positive nitrocellulose membrane (Roche, Indianapolis, IN, USA) by capillary transfer. The membrane was hybridized with digoxigenin-labelled terminal restriction fragment (TRF) telomere specific probe which was detected with CDPStar (Roche, Indianapolis, IN, USA) using X-ray film (Kodak, Rochester, NY, USA). The TRF telomere digoxigenin-labelled probe was prepared by PCR. Primers specific for the telomere sequence F: (CCCTAA)_4_, R: (TTAGGG)_4_ were amplified by non-template PCR (94°C/1.5 min, 94°C/45 s, 52°C/30 s, 72°C/1 min, 72°C/10 min, 30 cycles).The films were scanned with a ScanMaker i800 (Microtek, Taiwan) scanner. Densitometry was performed using Image-Master VSD Software (Amersham Biosciences, UK).

### Statistical calculations

All statistical calculations and simulations were computed by Mathematica 6.0 software (Wolfram Research, Inc. UK).

## Supporting Information

Figure S1 **Distribution of telomere fluorescence signal relative differences between sister telomeres in MJ90 cells.** A) PD 42 and B) PD52.
(TIF)

Figure S2 **Arithmetic mean of relative differences with respect to longer telomeres in MJ90 cells.** A) PD42 and B) PD52.
(TIF)

Figure S3 **Arithmetic mean of absolute differences with respect to longer telomeres in MJ90 cells.** A) PD42, and B) PD52.
(TIF)

Figure S4 **Standardized residuals for the regression models.** A) PD32 and B) PD42 and C) PD 52.
(TIF)

Figure S5 **Bootstrap samples of** ***T*** **statistics: histograms of the bootstrap sample** T* **(9) compared with standard normal p.d.f. (blue line, left) and the samples normal Q-Q plots (right).** A) PD32 B) PD42 and C) PD 52.
(TIF)

Figure S6 **Bootstrap samples of** ***T_0_*** **statistics: histograms of the bootstrap sample** T_0_* **(13-14) (left) compared with histograms of the standardized residuals (right).** A) PD32 B) PD42 and C) PD 52.
(TIF)

Figure S7 **Bootstrap samples of** ***Z*** **_σ_ statistics: histograms of the bootstrap sample** ***Z*** _σ_* (16) compared with standard normal p.d.f. (blue line, left) and the samples normal Q-Q plots (right). A) PD32 B) PD42 and C) PD 52.
(TIF)

Figure S8 **Estimated regression model: regression line (blue), 95% CIs of expected values of** ***Y*** **given** ***x*** **(ordinates of the green lines), and 95% CIs of the response values of** ***Y*** **given** ***x*** **(ordinates of the red lines).** A) PD42 and B) PD 52.
(TIF)

Table S1 Statistics of the model validation.
(DOCX)

Data S1 **Supporting Methods and References.**
(DOCX)
