# Supplementary figures and images for: Telomere Q-PNA-FISH - Reliable Results from Stochastic Signals
Source: PLoS One. 2014 Mar 18;9(3):e92559. doi: 10.1371/journal.pone.0092559 (PMC3958560; doi:10.1371/journal.pone.0092559)

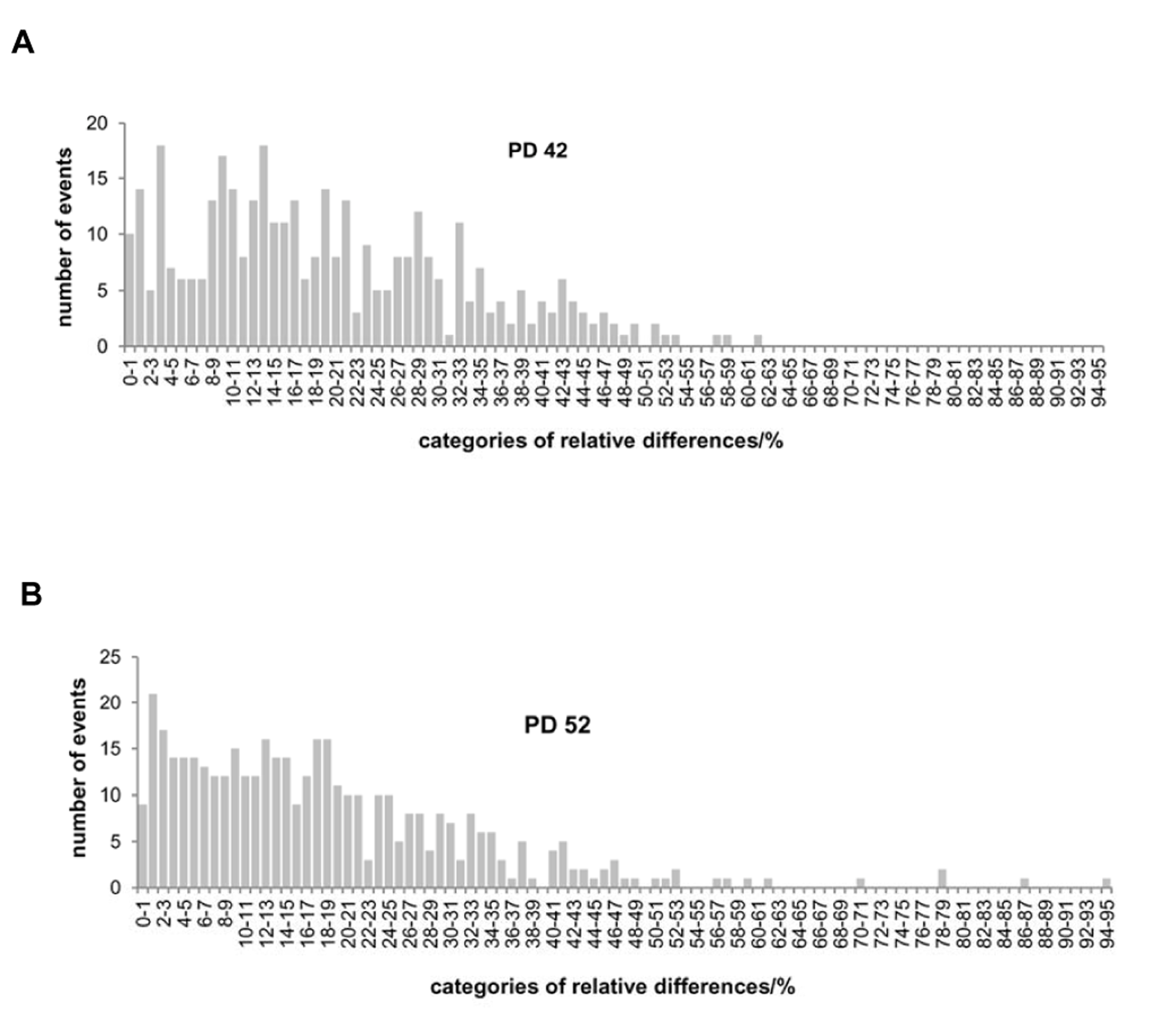

Supplement: Figure S1 — Distribution of telomere fluorescence signal relative differences between sister telomeres in MJ90 cells. A) PD 42 and B) PD52. (TIF) [file pone.0092559.s001.tif]

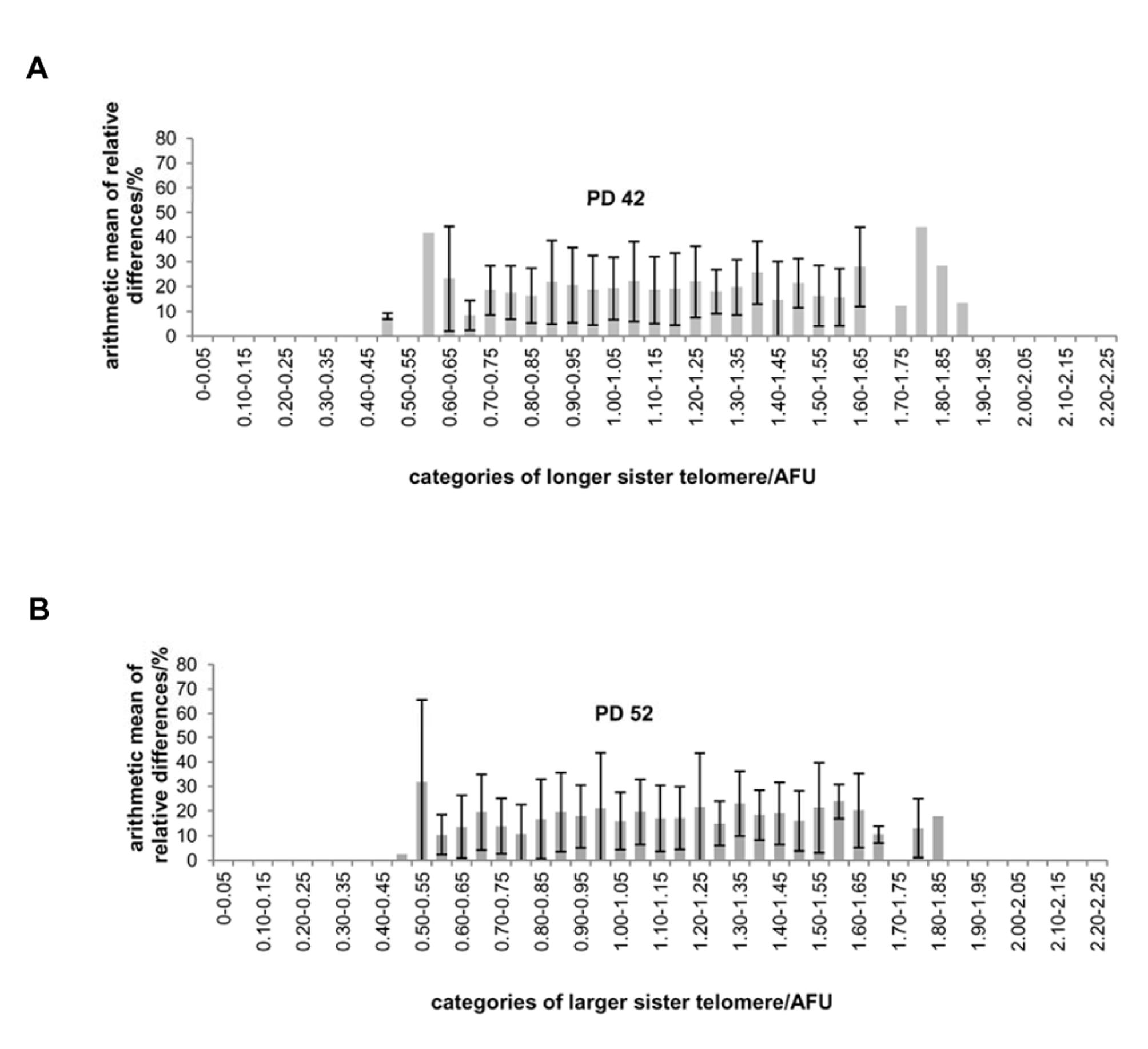

Supplement: Figure S2 — Arithmetic mean of relative differences with respect to longer telomeres in MJ90 cells. A) PD42 and B) PD52. (TIF) [file pone.0092559.s002.tif]

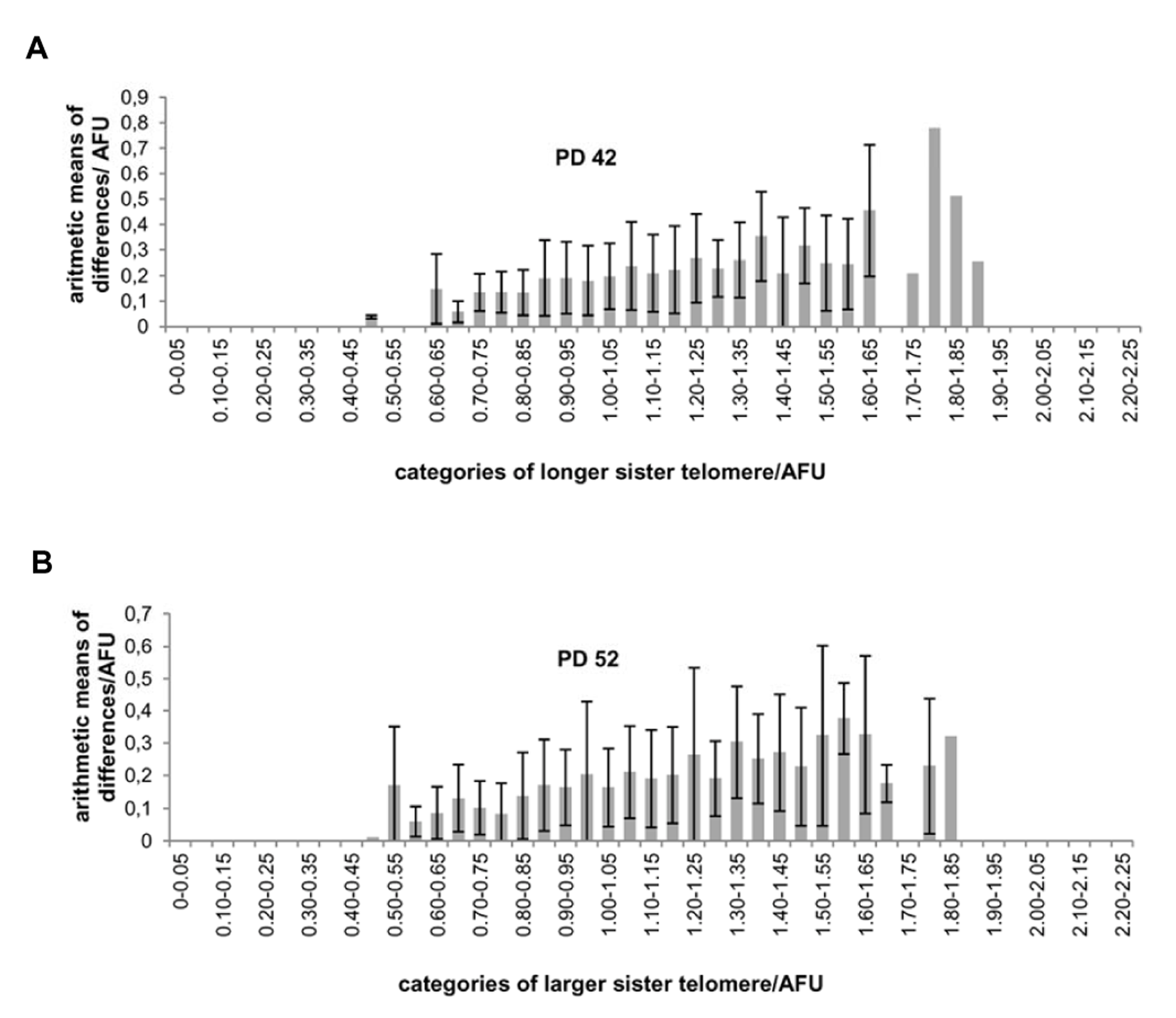

Supplement: Figure S3 — Arithmetic mean of absolute differences with respect to longer telomeres in MJ90 cells. A) PD42, and B) PD52. (TIF) [file pone.0092559.s003.tif]

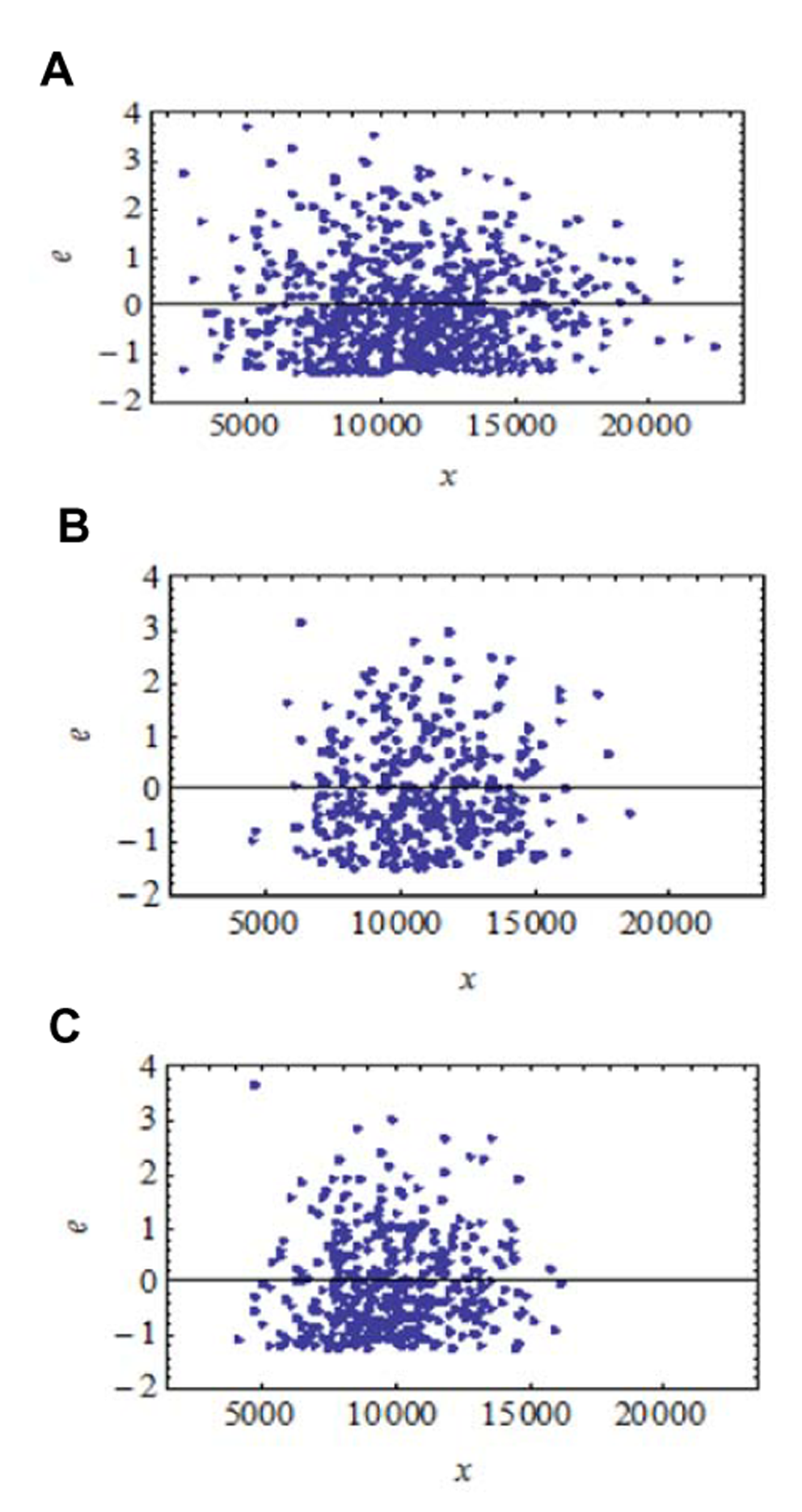

Supplement: Figure S4 — Standardized residuals for the regression models. A) PD32 and B) PD42 and C) PD 52. (TIF) [file pone.0092559.s004.tif]

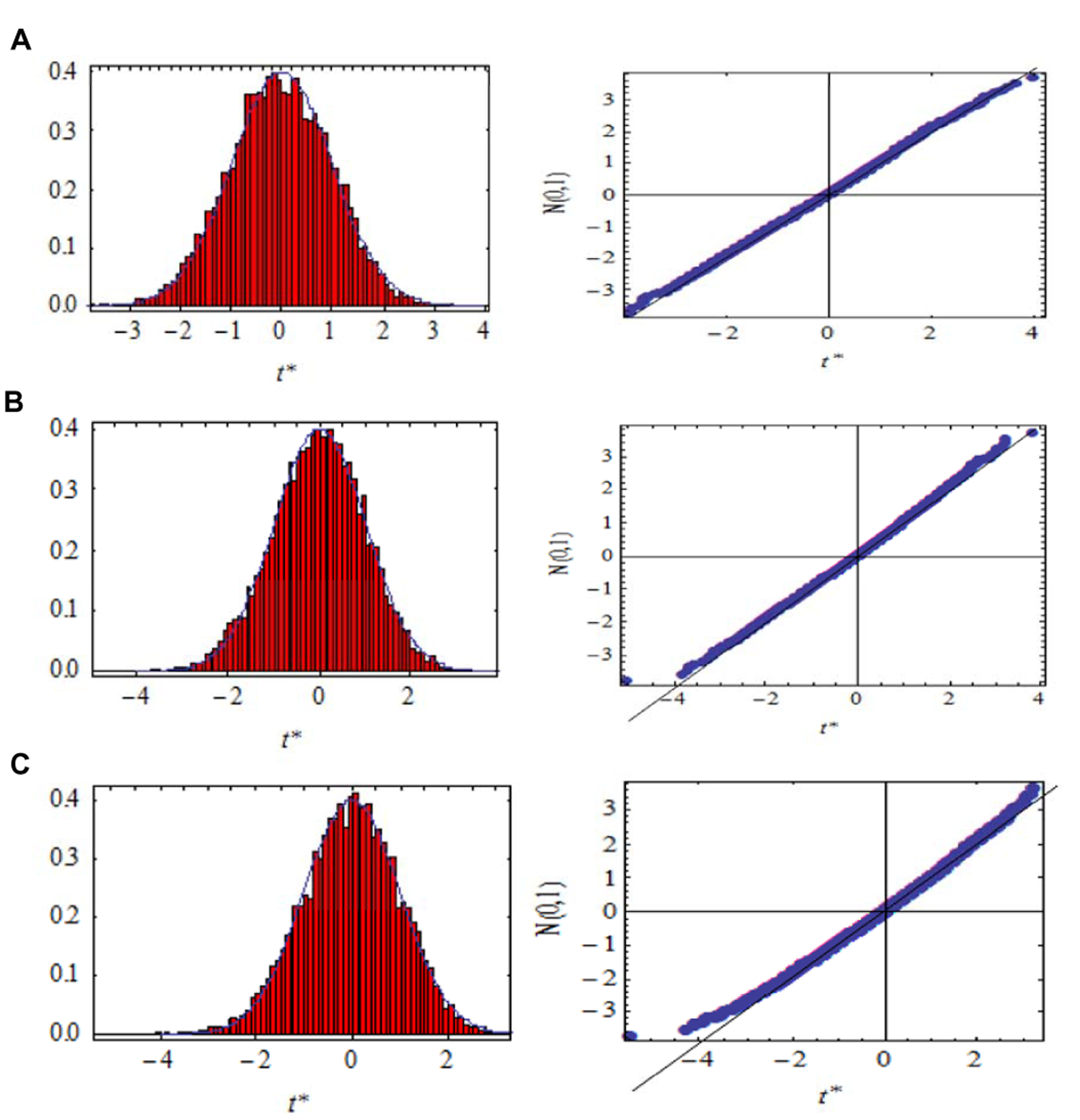

Supplement: Figure S5 — Bootstrap samples of T statistics: histograms of the bootstrap sample T* (9) compared with standard normal p.d.f. (blue line, left) and the samples normal Q-Q plots (right). A) PD32 B) PD42 and C) PD 52. (TIF) [file pone.0092559.s005.tif]

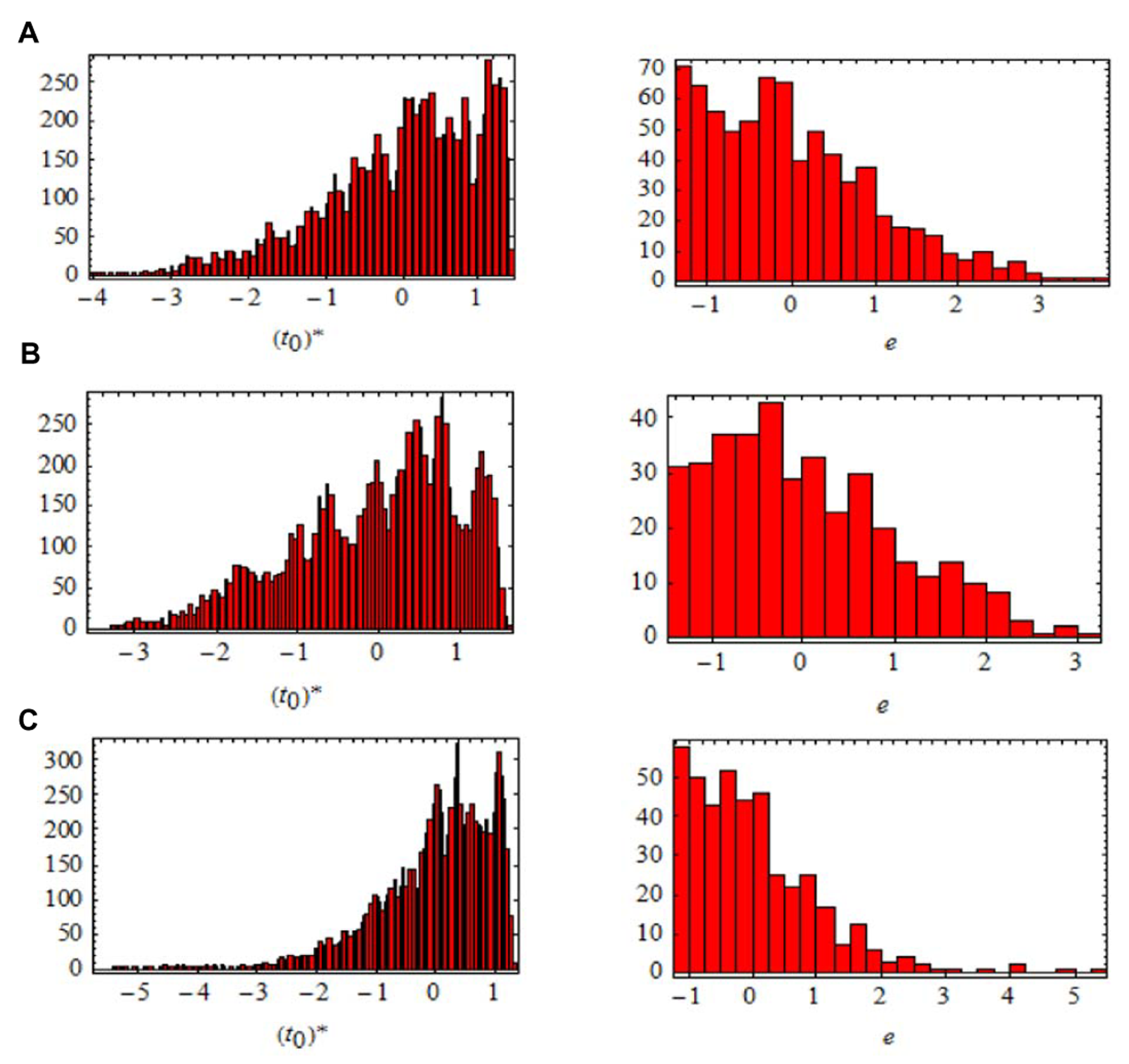

Supplement: Figure S6 — Bootstrap samples of T0 statistics: histograms of the bootstrap sample T0* (13-14) (left) compared with histograms of the standardized residuals (right). A) PD32 B) PD42 and C) PD 52. (TIF) [file pone.0092559.s006.tif]

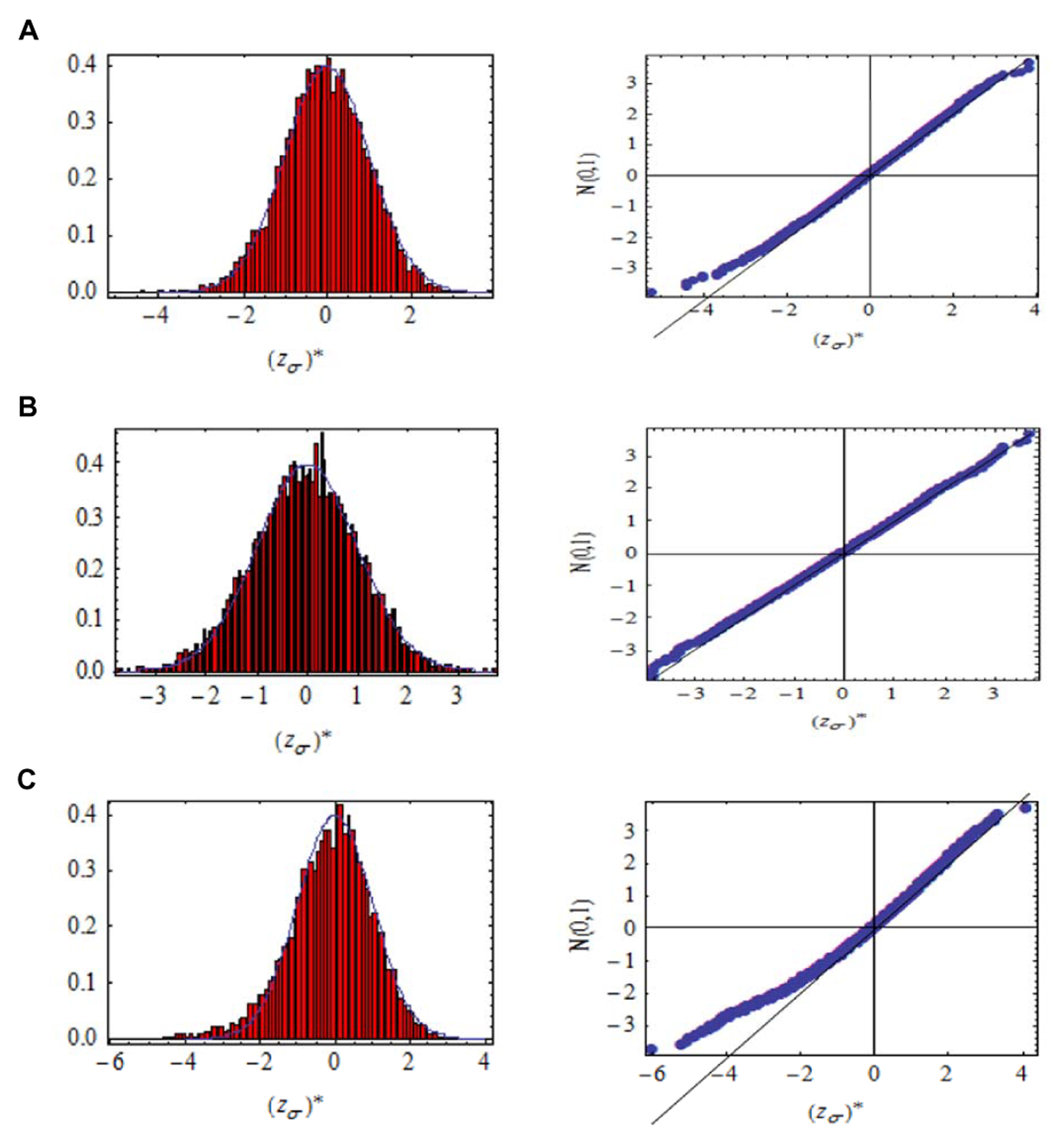

Supplement: Figure S7 — Bootstrap samples of Z σ statistics: histograms of the bootstrap sample Z σ* (16) compared with standard normal p.d.f. (blue line, left) and the samples normal Q-Q plots (right). A) PD32 B) PD42 and C) PD 52. (TIF) [file pone.0092559.s007.tif]

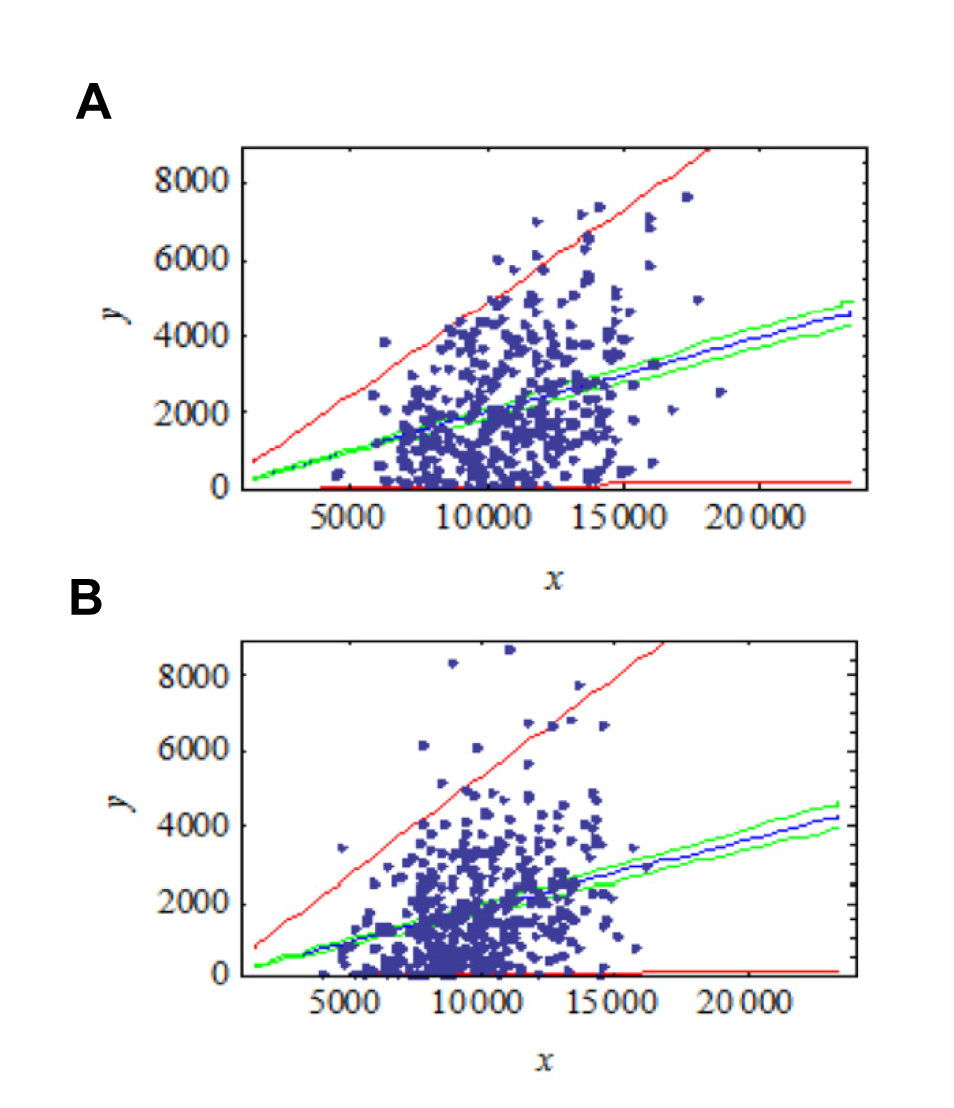

Supplement: Figure S8 — Estimated regression model: regression line (blue), 95% CIs of expected values of Y given x (ordinates of the green lines), and 95% CIs of the response values of Y given x (ordinates of the red lines). A) PD42 and B) PD 52. (TIF) [file pone.0092559.s008.tif]
