# Supplementary material for: Telomere Q-PNA-FISH - Reliable Results from Stochastic Signals
Source: PLoS One. 2014 Mar 18;9(3):e92559. doi: 10.1371/journal.pone.0092559 (PMC3958560; doi:10.1371/journal.pone.0092559)
Supplement: Table S1 — Statistics of the model validation. (DOCX) [file pone.0092559.s009.docx]

|  | | Points in 95% CI-all-points | |  | Points in 95% CI-all-but-one-point | |
| --- | --- | --- | --- | --- | --- | --- |
| MJ90 | Sample size (*n*) | Frequency (*f*) | Relative Freq. (*r* %) |  | Frequency (*f*) | Relative Freq. (*r* %) |
| PD 32 | 742 | 706 | 95.6 |  | 704 | 94.9 |
| PD 42 | 379 | 360 | 95.0 |  | 359 | 94.7 |
| PD 52 | 423 | 404 | 96.2 |  | 405 | 95.7 |

**Supplementary Table 1:** Statistics of the model validation
