# Supplementary material for: Telomere Q-PNA-FISH - Reliable Results from Stochastic Signals
Source: PLoS One. 2014 Mar 18;9(3):e92559. doi: 10.1371/journal.pone.0092559 (PMC3958560; doi:10.1371/journal.pone.0092559)
Supplement: Data S1 — Supporting Methods and References. (DOCX) [file pone.0092559.s010.docx]

**Supplementary Data**

**Telomere Q-PNA-FISH - Reliable Results from Stochastic Signals**

**Andrea Cukusic Kalajzic**^1^**, Nikolina Skrobot Vidacek**^1^**, Miljenko Huzak**^2^**, Milena Ivankovic**^1^**, Ivica Rubelj**^1*^

Affiliation: ^1^Department of Molecular Biology, Ruder Boskovic Institute, 10000 Zagreb, Croatia.

Affiliation: ^2^Department of Mathematics, University of Zagreb, 10000 Zagreb, Croatia.

*Correspondence: Ivica Rubelj, Department of Molecular Biology, Ruder Boskovic Institute, Bijenicka cesta 54, 10000 Zagreb, Croatia. Tel: +385 1 4561 093; fax: +385 1 4561 177; e-mail: rubelj@irb.hr

## Supporting Methods

## Statistical model

Distribution of telomere length difference between chromosome sisters can simply be modeled in the following way.

Let (*X*,*Y*) represent a pair of sister chromatids, where *X* is the length of the longer telomere (i.e. longer sister), and *Y* is the difference between lengths of the longer and shorter telomeres (i.e. telomere length difference between longer and shorter sisters). For every pair, the relative difference in telomere lengths between the sisters is a number *Z* = *Y* / *X*, with 0 < *Z* < 1 necessary. More precisely, let

(1) $\left(X_{1},Y_{1}\right),\left(X_{2},Y_{2}\right),\ldots,\left(X_{n},Y_{n}\right)$(

be *n* pairs of chromosome sisters in the sample (of a homogeneous data), (*X_i_*,*Y_i_*) representing the *i*th pair, *i* = 1, 2, …, *n*, and let

(2) $Z_{1}=\frac{X_{1}}{Y_{1}},Z_{2}=\frac{X_{2}}{Y_{2}},\ldots,Z_{n}=\frac{X_{n}}{Y_{n}}$ be their relative differences in telomere lengths. We assume that the data sets in this paper, named as “PD 32”, “PD 42” or “PD 52”, are homogeneous, in a way as explained in the following sentences.

Since the origin cell of any pair of sisters is chosen randomly and the splitting process resulting in a pair of chromosome sisters is independent of the other cells and the other chromosomes in the cell, we may think of the sample (1) as a sequence of independent random vectors. By examining the empirical data (Figure 1 and Figures S1A and S1B) we notice a significant degree of variability among the values of (2) for any of data sets. Consequently, (2) is a sequence of random (and by the properties of (1), independent) variables.

Our goals are to estimate their mean value and by using it, to predict the telomere (absolute) differences between sisters for a given telomere length of the longer sister. To do that, we need a proper statistical model for (1).

By examining the category plots of *Y*-values with respect to the categories (intervals) of *X*-values for each data sets (Figure 4 and Figures S3A and S3B), we notice that the conditional means and standard deviations of *Y* w.r.t. to the (categories) of *X*  linearly depend on the *X*-category means. Hence, it seems plausible to propose the following regression model for (1):

(3) $Y_{i}=a\cdot x_{i}+x_{i}\cdot E_{i},$ $i=1,2,\ldots,n$

where *x_i_*  represents a value of *X_i_* for all *i* = 1, 2, …, *n*, *a* is a regression parameter (representing the mean relative difference in telomere lengths between sisters), and

(4) $E_{1},E_{2},\ldots,E_{n}$

is a sequence of independent and equally distributed random variables with mean value 0 and

common standard deviation *σ* > 0, representing random errors. By this model, random variables (2) are independent and identically distributed with mean *a* and variance *σ*^2^ conditionally on *X*_1_ = *x*_1_ *, X*_2_ = *x*_2_, …, *X_n_* = *x_n_*, and *Y_i_* is distributed with mean

(5) $\text{E}\left[Y|X=x_{i}\right]=a\cdot x_{i}$

and variance

(6) $\text{Var}\left[Y|X=x_{i}\right]=\sigma^{2}\cdot x_{i}^{2},$

conditionally on *X_i_* = *x_i_*, *i* = 1, 2, …, *n*.

## Statistical estimation of telomere length difference

For estimating parameters *a* and *σ*^2^ we use the method of weighted least squares (WLS) with criterion function

(7) $L(a)=\sum_{i=1}^{n} \frac{1}{x_{i}^{2}}(Y_{i}-a\cdot x_{i})^{2}.$

We obtained the following unbiased estimators of *a* and *σ*^2^ by minimizing the criterion (7):

(8) $\hat{a}=\frac{1}{n}\sum_{i=1}^{n} \frac{Y_{i}}{x_{i}}$, ,

i.e. the sample mean and variance of (2).

95% confidence interval for *a* can be constructed by using standardized version of random variable $\hat{a}$ as a pivot variable:

(9) .

If random errors (4) were normally distributed then *T* would have Student’s distribution. This is not the case for our data sets since the distributions of residuals (as approximations of random error distributions) seem to be highly asymmetric (right histograms in Figure S6). On the other hand *T* has standard normal distribution asymptotically when the sample size *n* goes to infinity by the central limit theorem (see e.g. Theorem 5 of Section 5 and Section 6 in [1]). The question is: are sample sizes of our data sets large enough that the approximations of distribution of *T* by the standard normal are satisfactory? Since we are not sure what the answer to this question is, we use the bootstrap approximation of the distribution of *T* as more precise method of distribution approximation [2].

Let *T** represent bootstrap version of *T*, and let *t**_0.025_ and *t**_0.975_ represent its 0.975 and 0.025 quantiles, respectively. Then approximate 95% confidence interval for *a* is:

(10)

To construct 95% confidence interval for expected value E [*Y* | *X* = *x*] = *ax* for any given value *x* of *X*, we use the standardized version of its point estimator $\hat{a}x$ as a pivot variable. Notice that for any *x* > 0 this variable equals *T* given by expression (9):

(11) .

Hence, the left and right sides of approximate (bootstrap) 95% confidence interval of E [*Y* | *X* = *x*] as functions of *x*, are

(12) ,

respectively.

For the construction of 95% confidence interval for response value *Y* = *ax* +*xE* when *X* = *x* is given (and *E* is independent of random errors (4)), a pivot variable is the standardized version of $\hat{a}\cdot x-Y$:

(13) .

We approximate the distribution of *T*_0_ by the bootstrap method too. Let

(14)

be its bootstrap version, and let (*t*_0_)*_0.025_ and (*t*_0_)*_0.975_ represent its 0.975 and 0.025 quantiles, respectively. Then the left and right sides of approximate (bootstrap) 95% confidence interval of *Y* for given *X* = *x*, as functions of *x*, are

(15) ,

respectively. Notice that histograms of bootstrap samples of *T*_0_ (Figure S6) have the same shape (but with opposite frequency order) as histogram of corresponding standardized residual since the distributions of *T*_0_ are convolutions of the distributions of $\hat{a}$ (with the standard deviations equal to $\sigma/\sqrt{n}$) and *Y* / *x* (with the standard deviations equal to ), as expected by definition (13) of *T*_0_.

Finally, we need to estimate 95% confidence interval of the standard deviation of the errors . For a pivot variable we use the standardized version of statistic 2 log$\hat{\sigma}$:

(16) ,

where $\hat{\kappa}$is the sample kurtosis. It can be proved that *Z* has standard normal distribution asymptotically when the sample size *n* goes to infinity by using asymptotic normality of the sample variance and Cramér theorem applied on logarithmic function (Example 3 and Theorem 7 of Section 7 in [1]). By the same reasons as in the case of statistic *T* we use the bootstrap approximation of the distribution of *Z*.

Let *Z** represent bootstrap version of *Z*, and let *z**_0.025_ and *z**_0.975_ represent its 0.975 and 0.025 quantiles, respectively. Then approximate 95% confidence interval for is:

(17)

In all cases the bootstrap distributions are approximated by Monte Carlo method: bootstrap samples of size *M* = 9999 are taken by simple random sampling method with replacements from empirical distributions based on observed samples (2). Then 0.025 and 0.975 quantiles are estimated by 250^th^ and 9750^th^ order statistics based on appropriate (ordered) bootstrap sample. All statistical calculations and simulations are obtained by Mathematica 6.0 software (Wolfram Research, Inc.).

## Results of model estimation and validation

Estimates of the model parameters are presented in Tables 1 and 2 (see the paper).

The validity of the proposed model (3) for all our data sets can be justified, first, by the graphs of standardized residuals (Figure S4) which show high reliability of hypothesis (6) about error variances in the model for all data sets.

The second argument in favor of the proposed model and the estimation method is the fact that in all cases not more than 5% of data points lie outside of the areas between the red lines (Figure 5 and Figure S8) that are representing the graphs of left and right sides (15) of 95% confidence intervals [ly(*x*), ry(*x*)] of *Y* for a given *X* = *x*. Briefly, we call such confidence intervals CI-all-points (see columns 3 and 4 of Table S1) that provide frequencies (*f*) and relative frequencies (*r %*) of points whose *y*-values are inside of the corresponding CI-all-points). At the same figures, blue line represents the regression curves *x* E[*Y* | *X* = *x* ], and green lines represent the left and right sides (12) of 95% confidence intervals [lm(*x*), rm(*x*)] of E[*Y* | *X* = *x* ].

The third validation method used is Leave-one-out Cross Validation (LOOCV, [3]). For a given data set, and for all *i* = 1,2,…, *n*, we omit *i*-th point (*x_i_,y_i_*) from the data set and using (other) *n* – 1 points we estimated 95% confidence intervals with sides (15) for prediction of response *Y* at *X* = *x_i_* by the proposed method of estimation (briefly: CI-all-but-one-point). Statistic *f* in column 5 of Table S1 represents the total number of points (*x_i_, y_i_*) whose *y*-values *y_i_* lie inside of corresponding 95% CI-all-but-one-point (*r* % in column 6 of Table S1 represents the relative frequency of these points in the data set). Notice the fact that in all cases about 5% of points do not lie inside of corresponding predicted 95% confidence intervals which show high predictive ability of the proposed model and the estimation method.

## Comparison of the parameters between different groups

Histograms of bootstrap samples (Figures S5 and S7) of random variables *T* (9) and *Z* (16) in all cases are very good approximations of standard normal curve (q-q plots Figures S5 and S7). Hence, to test the significance of hypotheses that the mean difference between sister telomeres with smaller PD (*a*_1_) is greater than the mean difference between sister telomeres with greater PD (*a*_2_) (H_1_ : *a*_1_ > *a*_2_) , we can assume that the usual *z*-test statistic,

(18) ,

under neutral hypothesis (H_0_ : *a*_1_ = *a*_2_) is approximately normally distributed with zero mean and unity variance. The results of appropriate one-sided tests are in Table 3.

Similarly, we can assume that statistics

(19) ,

under neutral hypothesis that the standard deviations between two groups with different PDs are equal (H_0_ : _1_ = _2_), is approximately normally distributed with zero mean and unity variance. Since in some cases q-q plots (right graphs at Figure S7) show that the bootstrap samples of statistics *Z* have fatter left-tails we calculated *p*-values (denoted by *p**) based on bootstrap approximations of test statistics *Z*_12_. The results of appropriate two-sided tests are in Table 4.

In cases of both statistics *Z*_12_ and *Z*_12_ their normality’s are consequences of the asymptotic properties of the statistics (9) and (16), consistency of the sample standard deviations and kurtosis, normality of the simulated data, independency of the samples, and Slutsky theorems (see e.g. Theorem 6 of Section 6 in [1]).

**Supporting references**

1. Ferguson TS (1996) A course in large sample theory. London; New York: Chapman & Hall.

2. Davison AC, Hinkley DV (1997) Boostrap methods and their applications. New York: Cambridge University Press.

3. Stone M (1974) Cross-Validatory Choice and Assessment of Statistical Predictions. J R Stat Soc Ser B Methodol 36: 111–147. doi:10.2307/2984809.
